# Enhancing the nutritional value of date palm leaves via solid-state fermentation and its impact with single-cell protein on lambs’ performance

**DOI:** 10.1007/s11250-026-05094-7

**Published:** 2026-06-01

**Authors:** Hany M. Gado, Faisal S. Al Barakeh, Zeinab R. Mohammed, Hend A. Sayed, Abdelfattah Z. M. Salem

**Affiliations:** 1 Faculty of Agriculture, Ain Shams University, P.O. Box 68, Hadayek Shoubra, Cairo, 11241 Egypt; 2 Barari Natural Resources, Raising Sheep in the Forest Project., Cairo, Egypt; 3 Dipartimento di Scienze del Suolo, della Pianta e degli Alimenti (Di.S.S.P.A.), Università degli Studi di Bari, Via Giovanni Amendola, 165/a, 70126 Bari, BA Italy

**Keywords:** Solid-state fermentation, Date palm leaves, Lambs, Single cell protein

## Abstract

The study looked at how solid-state fermentation affects date palm leaves (DPL) and how it influences lamb performance, both with and without single-cell protein. DPL was fermented using three different strains of microbes. Using the Cornell Net Carbohydrates and Protein System, the nutritional value of DPL treatments, including fermented date palm leaves (FDPL) and DPL supplemented with single-cell protein designated as high protein (HP), was assessed under the study for chemical composition. To assess FDPL, eighteen crossbred male lambs, with an average live weight of 25 ± 0.2 kg, were divided into two groups. Whereas the second group got 40% concentrate feed mix (CFM), 50% FDPL, and 10% HP, the first group was fed a meal comprising 40% CFM and 60% FDPL. Compared with unfermented DPL, FDPL increased crude protein by 60% and reduced crude fiber by 28.7%. The calculated total digestible nutrients (TDN) value increased from 38.7 to 46.8% (+ 21.0%). Supplementing FDPL with HP further increased crude protein from 8.0 to 15.8% (+ 97.5%) and increased TDN from 46.8 to 66.7% (+ 42.5%). In the feeding trial, HP supplementation increased crude protein, TDN, and metabolizable energy intake, improved nutrient digestibility, feed conversion ratio, and feed efficiency, increased final body weight and average daily gain. Serum total protein, globulin, urea, and glucose increased, whereas AST and ALT remained within the normal physiological range. Overall, solid-state fermentation improved the feeding value of DPL, and the addition of a protein-rich single-cell protein product to FDPL further enhanced lamb performance without evidence of adverse health effects.

## Clinical trial

Not applicable.

## Introduction

The use of agricultural by-products in ruminant feeding can reduce feed costs, improve resource efficiency, and lessen competition between human food and animal feed (Hussein et al. 2024). Date palm cultivation generates large amounts of fibrous residues, including leaves, fronds, petioles, racemes, and pedicles (EL-Mously 2023). Although these residues are widely available in date-producing regions, their use in animal feeding remains limited because of their low crude protein concentration, high structural carbohydrate content, and relatively high lignification (Mahrous et al. 2021; Hamdon et al. 2022). Consequently, untreated date palm leaves (DPL) are generally characterized by poor digestibility and modest nutritive value.

The nutritive value of lignocellulosic by-products can be improved through biological processing. Fermentation with fibrolytic microorganisms can partially degrade cell-wall components, increase soluble nutrients, and improve the accessibility of structural carbohydrates to ruminal microbes (Nyochembeng et al. 2008; Salem et al. 2013). In parallel, single-cell protein (SCP) has received increasing attention as a sustainable protein source because microbial biomass can supply crude protein, essential amino acids, vitamins, and other bioactive compounds (Ritala et al. 2017; Upadhyaya and Chandra 2024). Combining biological treatment of fibrous residues with SCP supplementation may therefore provide a practical strategy to convert low-value agricultural biomass into more useful ruminant feed.

Accordingly, the present study aimed to evaluate the effect of solid-state fermentation on the chemical composition and calculated nutritive value of DPL, and the effect of feeding FDPL with or without a commercial high protein supplement (HP) on nutrient intake, digestibility, growth performance, and selected blood metabolites in lambs.

## Materials and methods

Fronds of date palm were collected, and the leaves separated from the midrib and frond base were shredded by passing them through a mechanical shredder. Leaves were cut into small pieces of about 2 cm in length to prepare them for the fermentation process.

### Types of strains

This study included three strains of different microorganisms. Obtained from the Laboratory of Microbiology, Department of Microbiology, Ain Shams University, Egypt, *Aspergillus Oryzae* (EMCC Number: 163). Anaerobic bacterial strain *Ruminococcus flavefaciens* (DSM 25089-0118) was separated from rumen fluid taken from Ain Shams University, Egypt’s Animal Production Department. Lactic acid bacteria (*Lactobacillus plantarum*, DSM 26571) came from Ain Shams University’s Department of Microbiology. Among the numerous habitats these bacteria occupy are those of flora, human digestive tracts, animals, poultry, and insects. All strains were pure, showing exactly the same microscopic traits as known strains, and used in liquid form without any additions save for the corresponding growth medium for each strain.

Single-cell protein (SCP) taken from *Saccharomyces cerevisiae* and Bacillus sp. forms the HI-Pro™ (commercially produced with high protein, HP) formulation. This is a liquid that is easy to consume and contains a high amount of protein (almost 80% on dry mass basis) Some ingredients of this product (per 1 L): L-arginine (90,000 mg), lysine (75,000 mg), methionine hydroxy analogues (80,000 mg), vitamin B3 (5,000 mg), vitamin B5 (1,500 mg), and enough Milli-Q water to produce one liter, with no antibiotics added. Adding HP provides a protein source to improve the nutritional value and increase the protein content in food.

### Solid-state fermentation

Fronds were collected from a private palm farm with the approval of the farm owner, according to the established rules, without any harmful effect on the plants. The leaves separated from the midrib and the frond base were shredded by passing them through a mechanical shredder. Components of DPL were fermented in two steps. In the first step, 10 g of *Aspergillus Oryza* (10^10^ CFU) and 10 g of *Ruminococcus flavefaciens* (10^8^ CFU) were added to 1000 g DPL, and water was added to the components until the humidity became 45% (CFU= colony-forming units). This mixture was incubated at 30 °C for 24 h. In the second step, at the end of the incubation period, 4 g of *Lactobacillus Plantarum* (10^9^ CFU) and water were added to the components until the moisture content reached 60% and then incubated for 16 h at 37 °C. The mixture was then desiccated at 45 °C for 24 h according to the procedure described by Chen et al. (2010) and Mohammed et al. (2024). Five replicates were applied for every treatment in a completely randomized design (CRD).

### Chemical analysis

Based on AOAC (2006), a proximate analysis of feed material and its constituents was performed to determine dry matter (DM), crude protein (CP), ether extract (EE), crude fiber (CF), ash content, and the nitrogen free extract (NFE, with NFE = (100- (CP + EE + CF + Ash). The fiber proportion was determined by the method of Van Soest et al. (1991).

### Evaluation of the results with the CNCPS model

Cornell Net Carbohydrate and Protein System CNCPS v6.5 (Fox et al. 2004) is a mathematical model that was used to evaluate the nutritive value of DPL treatments and rations containing DPL and HP. The model was fed with all of the data and descriptions to evaluate the calculations of feed energy.

### Animal feeding trial

This study was approved by the Care of Experimental Animal and Research Ethics Committee of Ain Shams University, Agriculture Sector Committee (Approval No. 4-2025-16). The use of animals in our study was strictly in accordance with the directions for caring for experimental animals from the American Veterinary Medical Association (AVMA). The research was conducted at Sakha Experimental Station, Animal Production Research Institute, Agriculture Research Centre, Egypt. Animals were sacrificed according to the AVMA Guidelines for Euthanasia.

Eighteen crossbred male lambs (½ Finnish Landrace × ½ Rahmani) at 3 months of age (25 ± 0.2 kg, initial weight) were used in a completely randomized design. They were weighed, divided into two homogenous groups (9 lambs/group), and housed individually in a 60-day feeding trial. Animals received a diet containing 40% Concentrate mix (Table 1), animals in the first group were fed 60% fermented date palm leaves (FDPL), and those in the second group were fed 50% fermented DPL and 10% HP (FDPL + HP) mixed with concentrate mix before feeding. The animals were fed *ad libitum* twice a day at 8:00 and 15:00 to allow for the collection of orts, and they were randomly allocated to one of two experimental treatments. The animals weighing performed at start and every two weeks; final weight was recorded at day 60. The animals’ performance parameters calculated as follow:

Protein Efficiency Ratio (PER) = Total gain (kg)/Total CP intake (kg)

Energy efficiency = weight gain (kg)/ ME intake (Mcal).

CP efficiency = weight gain (kg)/ CP intake (kg).

DMI % BW = (Average daily DM intake ÷ Mean body weight over the feeding period) × 100; mean BW was calculated as the average of initial and final body weight per group.

RGR (%/day) = [(In (final weight) - In (initial weight))/days]*100, whereas: In: natural logarithm, whereas: In: natural logarithm.

Apparent digestibility coefficients were measured by the total fecal collection method. During the last 7 days of the feeding period (days 54–60), feces were collected daily from each individually housed animal, weighed, a representative sub-sample taken and stored at − 20 °C. Pooled samples per animal were thawed, mixed, and analyzed for DM, OM, CP, NDF and ADF. Apparent digestibility (%) = [(nutrient intake − nutrient in feces)/nutrient intake] × 100.

**Table 1:** Ingredient composition and chemical composition of the concentrate feed mixture

Ingredient	Percentage (%)
Crushed corn (maize)	30.0
Sunflower meal	6.0
Corn germ meal	5.0
Glutofeed 16%	16.0
Soybean hulls	18.8
High Pro powder	1.0
Wheat bran	20.4
Mineral and vitamin mix	0.2
Sodium bicarbonate	0.3
Magnesium oxide	0.1
Limestone (CaCO3)	1.2
Salt (NaCl)	0.8
Feed additive (“Fido”)	0.2
**Chemical component**:	**Value**
Dry matter (%)	90.00
Crude protein (% DM)	15.55
Crude fiber (% DM)	9.76
Ether extract (% DM)	2.97
Ash (% DM)	7.10
Nitrogen-free extract (% DM)	64.62
Total digestible nutrients (% DM)	68.75
Metabolizable energy (Mcal/kg DM)	2.41

### Blood collection and analysis

Six animals from each group had their jugular veins sampled 4 h after breakfast, once at the end of the feeding trial, and the samples were centrifuged for 20 min at 3000 rpm. For further examination, the supernatant was collected, frozen, and kept at -20 °C. Blood serum was analyzed for total protein, albumin, urea, glucose, amino aspartate transaminase (AST) and alanine transaminase (ALT) were determined colorimetrically, using kits available commercially (Biodiagnostic, Dokki, Giza, Egypt). Globulin concentration was estimated by subtracting the albumin concentration from the equivalent total protein concentration.

### Statistical examination

The experimental data were analyzed using one-way ANOVA (SPSS V.20) to determine the differences between treatments. Prior to analysis, assumptions of normality and homogeneity of variance were checked using the Shapiro–Wilk and Levene’s test, respectively. Pairwise comparisons were performed and means separated using Duncan’s multiple range tests and analysis of variance. Significant differences between treatments were determined at *P* < 0.05.

$${Y_i}\, = \,\mu \, + \,{T_i} + {e_{ij}}$$

Where, Y_i_: dependent variable; µ: overall mean; T_i_: The effect of treatment.

e_ij_: experimental error.

## Results

### Solid-state fermentation of date palm leaves

Solid-state fermentation improved the chemical profile of DPL (Table 2). Relative to unfermented DPL, FDPL increased CP from 5.0 to 8.0% DM and reduced CF from 44.9 to 32.0% DM. Fiber fractions also declined, with NDF, ADF, and ADL decreasing from 66.3, 46.0, and 23.5 to 54.5, 38.0, and 15.5% DM, respectively. The addition of HP further improved the chemical composition of both unfermented and fermented DPL, and the combination FDPL + HP produced the highest CP concentration and the lowest fiber fractions among all treatments.

**Table 2:** Chemical composition of date palm leaves and fermented date palm leaves with or without HP supplementation (% DM)

Item	DPL	DPL + HP	FDPL	FDPL + HP	SEM	*P*-value
Crude protein	5.0d	13.1b	8.0c	15.8a	0.30	< 0.001
Crude fiber	44.9a	40.6b	32.0c	21.5d	0.40	< 0.001
Ether extract	1.4c	1.3d	1.6a	1.5b	0.02	< 0.001
Ash	10.6a	9.6b	8.5c	6.9d	0.20	< 0.001
NDF	66.3a	59.9b	54.5c	43.5d	0.30	< 0.001
ADF	46.0a	41.6b	38.0c	28.5d	0.30	< 0.001
ADL	23.5a	21.3b	15.5c	6.9d	0.20	< 0.001

DPL: control date palm leaves, FDPL: fermented date Palm leaves with *Aspergillus oryzea+ Ruminococcus + Lactobacillus*, HP: high-protein single-cell protein supplement, NDF: neutral detergent fiber, ADF: acid detergent fiber, ADL: acid detergent lignin

The different superscripts (a, b) within the same row are significantly different (*P* < 0.05)

SEM: Standard error of the mean

### Addition of SCP to date palm leaves 

The CNCPS-derived nutritive values followed the same pattern as the chemical composition data (Table 3). Fermentation increased non-fibrous carbohydrates, soluble fiber, and TDN, and HP supplementation further improved the calculated energy value of the fermented material. The FDPL + HP treatment had the highest TDN, DE, ME, NEm, NEl, and NEg values among the evaluated DPL treatments.

**Table 3:** CNCPS-derived nutritive value of date palm leaves and fermented date palm leaves with or without HP supplementation

Item	DPL	DPL + HP	FDPL	FDPL + HP
Soluble fiber, %	15.1	16.0	25.0	31.4
Starch, %	2.1	2.1	2.1	2.1
Total CHO, %	76.0	76.0	75.5	75.8
NSC, %	2.1	2.1	2.1	2.1
NFC, %	16.1	17.0	25.8	32.4
TDN 1x, %	38.7	45.0	46.8	66.7
GE, Mcal/kg	3.8	3.9	3.9	4.1
DE, Mcal/kg	1.2	1.4	1.5	1.7
ME, Mcal/kg	1.1	1.15	1.2	1.5
NEm, Mcal/kg	0.6	0.7	0.8	1.4
NEl 3x, Mcal/kg	0.6	0.7	0.9	1.3
NEg, Mcal/kg	0.3	0.5	0.7	0.9

DPL: control date palm leaves, FDPL: fermented date Palm leaves with *Aspergillus oryzea+ Ruminococcus + Lactobacillus*, CHO, carbohydrates; NSC, non-structural carbohydrates; NFC, non-fibrous carbohydrates; TDN, total digestible nutrients; GE, gross energy; DE, digestible energy; ME, metabolizable energy; NEm, net energy for maintenance; NEl, net energy for lactation; NEg, net energy for gain

### Growth performance 

Lambs fed FDPL + HP had higher CP, TDN (8% and 8.5%, respectively) intakes than lambs fed FDPL alone, whereas concentrate intake, roughage intake, total DM intake, DMI as a percentage of body weight, and the TDN: CP ratio were similar between treatments (Table 4). The FDPL + HP diet also improved apparent digestibility of DM, OM, CP, NDF, and ADF and increased dietary TDN and ME concentrations (Table 5). These responses were accompanied by higher final body weight, total gain, average daily gain, feed efficiency, protein efficiency ratio, energy efficiency, CP efficiency, and relative growth rate, together with a lower feed conversion ratio.

**Table 4:** Feed and nutrient intake of lambs fed fermented date palm leaves with or without HP supplementation

Item	FDPL	FDPL + HP	SEM	*P*-value
Concentrate intake (kg/day)	1.00	1.00	0.02	1.000
Roughage intake (kg/day)	1.05	1.00	0.03	0.115
Total DM intake (kg/day)	1.95	2.00	0.04	0.229
CP intake (g/day)	190	205	4.50	0.004
TDN intake (kg/day)	1.40	1.52	0.03	0.001
ME intake (Mcal/day)	2.07	2.23	0.05	0.006
DMI (% of BW)	4.10	4.18	0.06	0.201
CP intake (% of BW)	0.40	0.43	0.01	0.008
TDN: CP ratio	7.37	7.41	0.09	0.663
ME intake per kg BW (kcal)	111.0	119.5	2.10	< 0.001

FDPL, fermented date palm leaves; HP, high-protein single-cell protein supplement; DM, dry matter; CP, crude protein; TDN, total digestible nutrients; ME, metabolizable energy; DMI, dry matter intake

SEM: Standard error of the mean

**Table 5:** Apparent digestibility, growth performance, and feed efficiency of lambs fed fermented date palm leaves with or without HP supplementation

Item	FDPL	FDPL + HP	SEM	*P*-value
**Digestibility and nutritive value**				
DM digestibility (%)	68.1	70.4	0.80	0.011
OM digestibility (%)	69.5	72.2	0.85	0.006
CP digestibility (%)	71.8	75.0	1.00	0.006
NDF digestibility (%)	63.3	66.4	1.10	0.012
ADF digestibility (%)	60.1	63.8	1.10	0.004
TDN (%)	71.8	74.3	0.75	0.004
ME (Mcal/kg DM)	2.3	2.4	0.03	0.004
**Growth performance and efficiency**				
Average initial weight (kg)	25.0	25.0	—	—
Final weight (kg)	39.5	42.2	0.45	< 0.001
Total gain (kg)	14.5	17.2	0.50	< 0.001
Duration (days)	60.0	60.0	—	—
Average daily gain (g/day)	242.0	287.0	8.0	< 0.001
Total DM intake (kg)	117.0	120.0	2.0	0.153
Feed conversion ratio	8.1	7.0	0.25	< 0.001
Feed efficiency (gain: feed)	0.12	0.14	0.005	0.001
Protein efficiency ratio	0.94	1.08	0.03	< 0.001
Energy efficiency (kg gain/Mcal)	7.0	7.7	0.2	0.003
CP efficiency (kg gain/kg CP)	76.3	83.9	2.0	0.002
RGR (%/day)	0.97	1.15	0.03	< 0.001

FDPL, fermented date palm leaves; HP, high-protein single-cell protein supplement; TDN, total digestible nutrients; ME, metabolizable energy; CP, crude protein; RGR, relative growth rate

SEM: Standard error of the mean

### Blood metabolites 

Compared with FDPL alone, feeding FDPL + HP increased serum total protein, globulin, urea, and glucose, whereas albumin, AST, and ALT were not materially affected (Table 6). Importantly, liver-enzyme activities remained within normal physiological limits, supporting the absence of an adverse health response.

**Table 6:** Blood metabolites of lambs fed fermented date palm leaves with or without HP supplementation

Item	FDPL	FDPL + HP	SEM	*P*-value
Total protein (g/dL)	6.6	6.9	0.12	0.031
Albumin (g/dL)	3.5	3.6	0.08	0.240
Globulin (g/dL)	3.2	3.4	0.07	0.017
Urea (mg/dL)	33.5	37.0	1.10	0.010
Glucose (mg/dL)	55.0	60.2	1.50	0.006
AST (U/L)	68.9	69.5	1.70	0.731
ALT (U/L)	25.5	25.2	0.60	0.628

FDPL, fermented date palm leaves; HP, high-protein single-cell protein supplement; AST, aspartate aminotransferase; ALT, alanine aminotransferase

SEM: Standard error of the mean

## Discussion

The main problems of DPL, as feed for ruminants, is low nutritional value, poor digestibility, high fiber with low protein, and limited utilization (Hussein et al. 2024). Previous studies show that while lowering fiber content, the fermentation of DPL improves the protein content of the treated DPL (Mahrous et al. 2021). The application of fibrolytic bacteria (*Ruminococcus flavefaciens* and *Aspergillus oryzae*) and the exogenous enzymes generated by microorganisms break down secondary compounds and fiber linkages in the feed, thereby increasing the digestibility of DPL. The findings complement Gado (2020), who observed that simple sugars, including cellulase and hemicellulase, can be efficiently transformed from complex sugars in plant materials, such as DPL, by means of enzymes generated by anaerobic bacteria. The DPL treated with additives may have a lower fiber content, due to microorganisms’ fermentation breaking down carbohydrates, which they utilize for growth and synthesis. High levels of neutral detergent fiber (NDF, 58%) make it difficult to ingest date palm byproducts, such as leaves, Hamdon et al. (2022). In our work, DPL’s fermentation lowered NDF relative to the control group to 48%. The improvement was evidenced by lower fiber levels and improved crude protein, which, after fermentation, resulted in a 30% increase in total digestible nutrients. These findings reflect what Sayed et al. (2023) discovered: Introducing *R. flavefaciens* bacteria to diets including palm kernel cake increased the breakdown of NDF and ADF. Fibrolytic bacterium *Ruminococcus flavefaciens* can break down the fibers in feed and increase the protein level by releasing some amino acids connected to the fibers during their breakdown and by boosting microbial protein during treatment. Low-quality by-products can be treated biologically to produce higher amounts of soluble fibers (soluble fibers and non-fiber carbohydrates; NFC = 100 − cNDF + CP+Fat + Ash) and lower amounts of fiber, thus improving the nutritional value of these by-products. The results match what Villas-Boas et al. (2002) found. According to Bennett and Faciola (2022), the best way to improve the nutritional value of food before eating is to add enzymes. Consistent with our results, Nalage et al. (2016) reported that in animal nutrition, single-cell protein is considered a great source of protein, carbohydrates, minerals, and vitamins.

### Addition of SCP to date palm leaves 

The CNCPS’s assessment of DPL’s enhanced nutritional value results from the combined actions of protein supplements and fermentation. While HP provides a significant increase in concentration of protein and other essential elements, digestibility and bioavailability are achieved by breaking down complex carbohydrates and fibers (Nalage et al. 2016). Higher crude protein and soluble fiber, explain the better feed conversion ratio of FDPL. These results are consistent with Olafadehan et al. (2014), who attributed shorter transit times in the gastrointestinal tract, improved palatability, acceptability, and consumption, enhanced rumen fermentation and digestibility in goats receiving bovine rumen content. This combination increases the whole nutritional value of FDPL, making it a more suitable feed for ruminants. Consistent with the results of the current study, Kholif et al. (2022a, b) demonstrated that ensiling DPL with multi-species probiotics or fibrolytic enzymes can improve feed efficiency and sheep lactation performance.

### Growth performance 

Compared to the FDPL group, HP greatly boosted the intake of protein, TDN, and ME. These increases slightly improved the TDN: CP ratio, which is used to evaluate the balance between energy and protein. The metabolizable energy intake per kilogram of body weight offers information on the energy density of consumption relative to animal size, which rises by 7.5% with the inclusion of HP. During the introduction of HP, nutrient digestibility was further improved, possibly due to the presence of microorganisms that facilitate digestion and metabolism; consequently, the fermentation process enhanced the nutrient digestibility of DPL. The fermentation of DPL (FDPL) improved the feed conversion ratio and average daily gain of lambs by about 13.6% and 18.5% compared with DPL, respectively. The supplementation of nutrient availability enhances the palatability and acceptability of the feedstuff and reduces anti-nutritional variables (Kholif et al. 2022a, b; Sayed et al. 2021). Superior growth performance and feed conversion efficiency in lambs follow from increased feed intake, digestion, and nutrient absorption. Furthermore, Mahrous et al. (2021) showed that increasing total mixed meals for lambs with a varied array of beneficial bacteria in the DPL improves growth performance, ruminal fermentation, voluntary feed intake, nutrient digestibility. Sayed et al. (2021) found that including *Ruminococcus flavefaciens* and other bacterial strains in feeds improved average daily gain and overall weight gain in lambs.

### Blood metabolites 

All the serum metabolites in this study lie within the normal physiological ranges (Kaneko et al. 2008; Yang et al. 1997). According to the blood analysis, there are no major health concerns, and all diets are supposed to satisfy the nutritional needs of the sheep. The observed increase in blood total protein and glucose levels in lambs fed FDPL + HP points to the higher crude protein and energy consumption. Particularly in the FDPL + HP group, higher blood protein levels point to increased protein metabolism and use, most likely due to better rumen digestion and microbial generation encouraged by the treated roughage and balanced concentrate mixture. This reflects the nutritional condition of the animal since Yang et al. (1997) found a positive link between blood protein concentrations and feed protein levels. There were no significant changes in liver function enzymes (ALT, AST), which shows that all diets were safe and well-accepted by the lambs, possibly indicating better liver function or less inflammation with this diet. Furthermore, glucose levels seemed to increase in line with TDN and ME intake, suggesting higher metabolic efficiency and energy availability. Lambs given FDPL with HP have improved blood metabolites that reflect metabolic activity and nutritional condition. Increased protein and carbohydrate metabolism is indicated by higher albumin and glucose levels (Feng et al. 2021). Even though the results were still normal, the increase in blood urea nitrogen (BUN) is probably due to eating more nitrogen, which means there might be too much protein that breaks down easily or not enough nitrogen being kept in the body. By increasing the nutritional value, fermentation and digestibility of the feed help lambs be healthier and more productive.

## Conclusion

Solid-state fermentation improved the feeding value of date palm leaves by increasing crude protein and reducing fiber fractions. In the present data set, fermentation increased CP by 60.0%, reduced crude fiber by 28.7%, and increased the calculated TDN value by approximately 21%. Supplementing the fermented material with HP further increased CP by 97.5% and TDN by 42.5%. In lambs, the FDPL + HP diet improved nutrient digestibility, average daily gain, and feed efficiency while maintaining normal liver-enzyme activities. These findings support the use of fermented date palm leaves, especially when combined with a protein-rich SCP supplement, as a promising feeding strategy for growing lambs.

## Data Availability

The datasets used and/or analysed during the current study available from the corresponding author on reasonable request.
